# Influence of early extensive posterior decompression on hyponatremia and cardiopulmonary dysfunction after severe traumatic cervical spinal cord injury: A clinical observational study

**DOI:** 10.1097/MD.0000000000021188

**Published:** 2020-07-17

**Authors:** Chaohua Yang, Gaoju Wang, Shuang Xu, Guangzhou Li, Qing Wang

**Affiliations:** Department of Orthopaedics, The Affiliated Hospital of Southwest Medical University, Sichuan China.

**Keywords:** cervical spinal cord injury, expansive decompression, hyponatremia, hypotension, laminoplasty, tracheotomy

## Abstract

Retrospective single institution observational study.

The aim of the present study was to analyze the influence of early extensive posterior decompression on complications in patients with severe traumatic cervical spinal cord injury (tcSCI).

Cervical SCI is associated with a high prevalence of hyponatremia and cardiopulmonary dysfunction. However, very few studies have focused on this exploration to reduce the incidence of SCI early complications.

We reviewed the medical records of consecutive patients undergoing extensive posterior decompression within 24 h for severe tcSCI (American Spinal Injury Association Impairment Scale [AIS] A to C) admitted between January 2009 and January 2018. The data collected retrospectively included age, gender, mechanism, and level of SCI, AIS grade, fracture or dislocation, electrolyte, and cardiopulmonary complications.

Of the 97 enrolled patients, the baseline AIS grade was AIS A in 14, AIS B in 31, and AIS C in 52. Improvement of at least two AIS grades was found in 26 (26.8%), and improvement of at least one grade was found in 80.4% of patients at discharge. Twenty-nine (29.9%) patients had mild hyponatremia, 8 (8.2%) had moderate hyponatremia, and 3 (3.1%) had severe hyponatremia during hospitalization. The incidences of hyponatremia, hypotension, and tracheotomy were 41.2%, 13.4%, and 6.2%, respectively. The mean forced vital capacity (FVC) on admission and at discharge was 1.34 ± 0.46 L and 2.21 ± 0.41 L (*P* < .0001), respectively. Five patients developed pneumonia.

Our results suggest that early expansive posterior decompression significantly reduces the incidence of hyponatremia, hypotension, and tracheotomy by promoting recovery of spinal cord function after severe tcSCI.

## 1 Introduction

Traumatic cervical spinal cord injury (tcSCI) causes dysfunction of the somatic and autonomic nervous system below the injury level, which not only causes paralysis but also causes various severe complications.^[1,2]^ Hyponatremia, hypotension, and pulmonary dysfunction are three major complications during the acute stage after tcSCI.^[3,4]^ These factors have been shown to be associated with worse outcomes, such as secondary SCI, and rarely with cardiac arrest, pulmonary edema, pneumonia, atelectasis, respiratory failure, and even death. Its treatment is usually more difficult and increases the financial burden.^[5–8]^ However, very few studies have focused on reducing the incidence of SCI early complications. Therefore, the purpose of this study is to explore the influence of early extensive posterior decompression on these acute stage complications in patients with severe tcSCI.

## 2 Materials and methods

### 2.1 Study design and patient population

The present study includes a retrospective cohort analysis of laboratory records for patients of a single institution. This study was approved by the hospital ethics committee in 2018 (ky20180105), and patient consent was obtained in selected patients. All consecutive individuals with severe tcSCI (AIS grade A–C) who underwent extensive posterior decompression (laminoplasty or laminectomy, with or without pedicle screw fixation) within 24 h after trauma from January 2009 to January 2018 were enrolled. Patients with complicating traumatic brain injury, operation history of cervical spine, tumors of other tissues and organs, or history of renal diseases were excluded.

Patient charts were reviewed with particular interest in age, gender, mechanism of injury, fractures or dislocations, time period from injury to operation, SCI level, operation procedures, AIS grade at admission and discharge, medications and preexisting medical condition, and hospitalization time.

### 2.2 Surgical intervention

**Figure 1:**
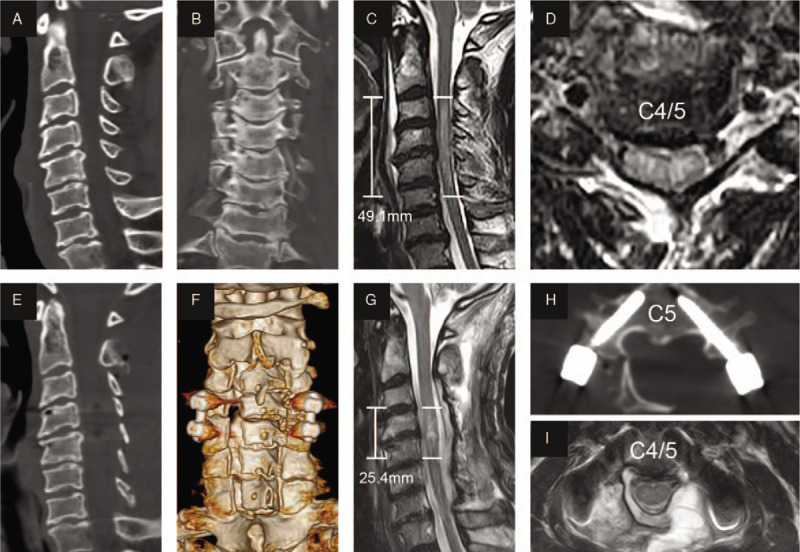
Representational preoperative (A–D) and postoperative (E–I) images of a 60-year-old male patient who sustained a spinal cord injury with posterior ligament injury. His AMS was 24 and AIS grade C. Midsagittal subaxial CT (A) indicated cervical spinal stenosis (CSS) from C3 to C6, and MRI (C) SCI with an intramedullary lesion length (IMLL) of 49.1 mm. The subarachnoid space (SAS) was absent at C4 and C5 (C and D). Laminoplasty of C3–7 and pedicle screw fixation of C4–5 were performed in this patient 13 h after trauma. Postoperative CT (E, F, and H) showed significant enlargement of the osseous spinal canal and good screw positioning. Postoperative MRI showed an IMLL of 25.4 mm (G) and successful decompression (G and H) indicated by the presence of an open anterior and posterior SAS 11 days after trauma. Fourteen days after injury, the patient recovered paralysis with an AMS of 52 and AIS grade D. The patient had moderate hyponatremia and no hypotension or tracheotomy during hospitalization.

**Figure 2:**
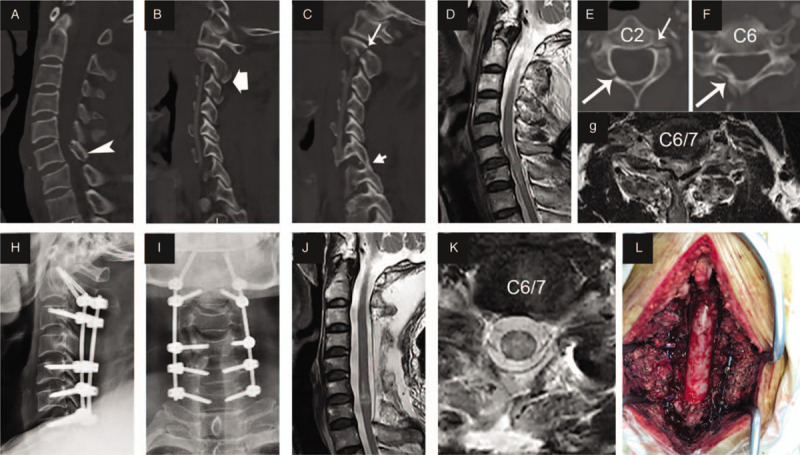
Representational preoperative (A–G), intraoperative (L) and postoperative (H–K) images of a 50-year-old male patient who sustained an atypical hangman fracture, C type fracture of C6 and spinal cord injury. His ASIA motor score (AMS) was 15 and AIS grade A. Sagittal subaxial CT indicated C6 translation rotation injury (A, arrowhead) and atypical hangman fracture (C and E, short arrow) accompanied by fracture of the right inferior articular process of C2 and C3 (B), locked facet of C6–7 (C, arrow), and right lamina fracture of C2 and C6 (E and F, long arrow). MRI (D and G) showed SCI at the C6 to C7 level, and the subarachnoid space (SAS) was absent at C6/7 (G). Laminectomy of C3–7 (L) and pedicle screw fixation from C2 to C7 were performed in this patient 10 h after trauma. Postoperative X-rays (H and I) showed good screw positioning and cervical sequence. MRI indicated successful decompression and opened SAS (J and K) 9 days after trauma. Fifteen days after injury, the patient recovered paralysis with an AMS of 29 and AIS grade B. The patient had mild hyponatremia and no hypotension or tracheotomy during hospitalization.

Decompression surgery was performed a mean of 16.5 ± 7.1 h (median 13.8; range 3.6–23.1 h) following trauma. All patients underwent posterior laminoplasty (Fig. 1) or laminectomy (Fig. 2), and pedicle screw fixation was performed simultaneously in the case of fractures and dislocations. Anterior surgery was added as a second stage only if neurological deterioration occurred due to a large nonreduced disc fragment identified on preoperative magnetic resonance imaging (MRI). After general anesthesia was successfully induced and orotracheal intubation was achieved, the patient was placed in the prone position, and continuous skull traction with a weight between 5 and 8 kg was applied using Gardner–Wells tongs to allow a maximally horizontal head position. The paravertebral muscles were detached from the spinous processes on both sides, and the processes were removed. The range of decompression was from at least one lamina above and below the edematous segment according to emergency MRI and/or absence of the subarachnoid space (SAS). Single open-door laminoplasty was performed using a high-speed air-burr drill. We usually dissect laminae on the severely paralyzed side and make hinges on the other side. When the lamina is fractured, laminectomy is performed. For patients with fractures and dislocations, reduction and pedicle screw fixation were performed using a previously described method.^[9,10]^ To relieve spinal cord compression as soon as possible, we usually implement laminoplasty or laminectomy first, insert the pedicle screw to recover cervical alignment, and then keep the door open with centerpiece plates or threads.

### 2.3 Hyponatremia assessment

Electrolyte detection and electrocardiography (ECG) monitoring were performed for all patients with severe tcSCI. Electrolyte tests were performed once a day for the first 3 days after admission and then every three days until no hyponatremia was detected twice. Hyponatremia was defined as a serum sodium concentration below 135 mmol/L in 2 consecutive blood samples. The patients were classified as having mild (*S*_Na+_ = 130–135 mmol L^−1^), moderate (*S*_Na+_ = 125–130 mmol L^−1^), or severe (*S*_Na+_ < 125 mmol L^−1^) hyponatremia. For patients with hyponatremia, we supplemented hypertonic saline at a rate of serum sodium concentration rise by no more than 8 mmol L^−1^ per day (excessively rapid correction of hyponatremia may lead to osmotic demyelination syndrome)^[11,12]^ until 140 mmol L^−1^ was reached.

### 2.4 Cardiovascular and pulmonary function assessments

Arterial blood pressure (BP) and heart rate (HR) were continuously monitored 24 h day^−1^ for 3 to 5 days after admission and then once a day until discharge. Patients with arterial hypotension, arterial systolic BP < 90 mm Hg and bradycardia < 50 min^−1^ required 0.5 mg of atropine for low HR and dopamine for hypotension. The forced vital capacity (FVC) was measured on admission and at discharge using a spirometer. Data on pulmonary infection and tracheotomy were collected.

### 2.5 Statistical analysis

Statistical analysis was performed using SPSS Statistics 19.0 (IBM, New York, NY). We used the mean ± standard deviation (SD) for continuous variables and absolute and relative frequencies for categorical variables. Independent samples *t* test was used for the statistical analyses of parametrically distributed variables. Fisher's exact test and chi-square test were used for categorical variables. A *P* value of <.05 was considered statistically significant, and all tests were two-tailed.

## 3 Results

### 3.1 Clinical data

In the period from January 2009 to January 2018, a total of 97 patients (male: 81; female: 16) met the eligibility criteria. The demographic and clinical data are presented in Table 1. Among the 97 patients initially enrolled in the trial, the baseline AIS grade was AIS A in 14, AIS B in 31, and AIS C in 52. Improvement of at least two AIS grades was found in 26 (26.8%) patients, and improvement of at least one grade in was found in 78 (80.4%) patients at discharge (Table 2). For the 88 patients (9 deaths were excluded) who were discharged to a rehabilitation center, the mean hospital length of stay was 16.6 ± 4.5 days (median 15.2; range 7.3–46.3 days). Nine in-hospital deaths were noted, including 3 patients who underwent tracheostomy. Sudden oxygen desaturation resulted in 1 death; hypotension and cardiac arrest resulted in 2 deaths; and malignant hyponatremia occurred in 1 case.

**Table 1:**
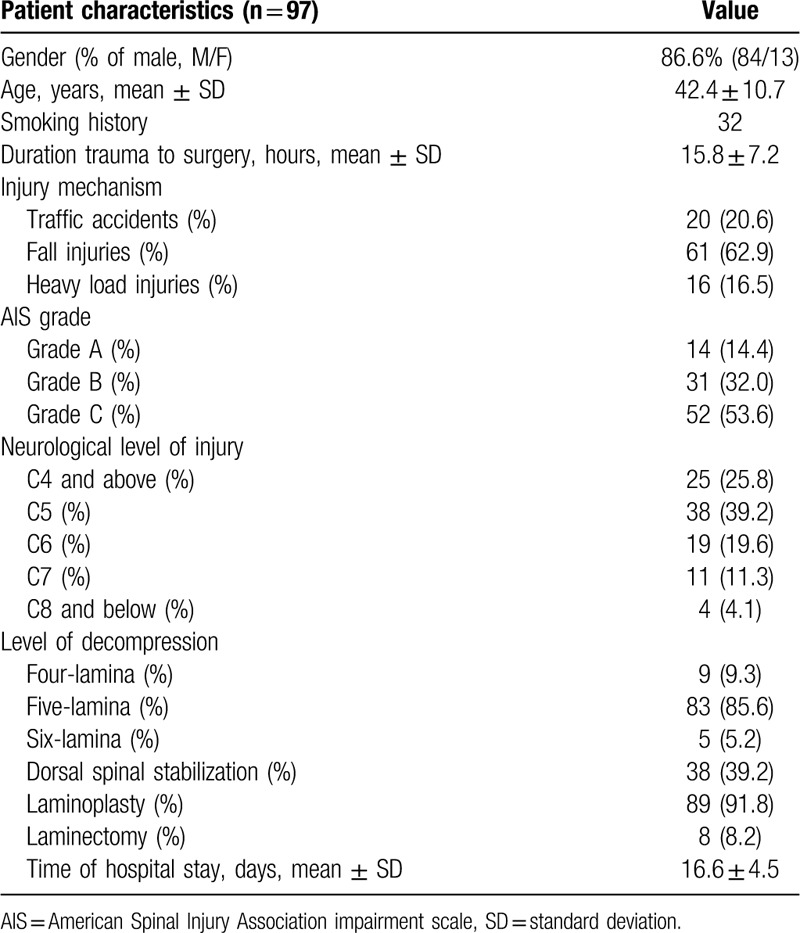
Epidemiological and clinical data.

**Table 2:**
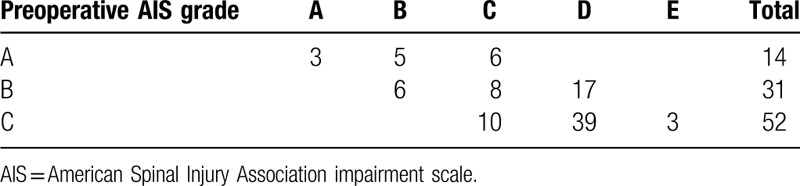
Changes in AIS grade from pre-operative to discharge.

### 3.2 Hyponatremia and cardiovascular and pulmonary function assessment results

Of the 97 enrolled patients, 29 (29.9%) had mild hyponatremia, 8 (8.2%) had moderate hyponatremia, and 3 (3.1%) had severe hyponatremia during hospitalization. Severe hyponatremia is more likely to occur in patients with an AIS grade of A or B, while patients with an AIS grade of A or B are more prone to exhibit hyponatremia (Table 3). In 71.4% of patients, hyponatremia was corrected within 10 days, whereas in 4 patients, it was corrected over 20 days.

**Table 3:**
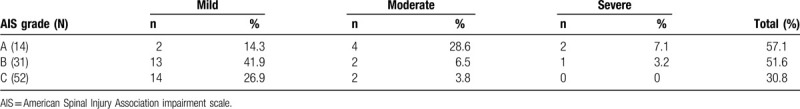
Hyponatremia and AIS grade.

Nineteen of the 97 patients had an average daily HR of <60 min^−1^, and 6 had episodes during which their HR was < 50 min^−1^, which required an intravenous injection of atropine. Thirteen patients with BP < 90 mm Hg required a dopamine pump ranging from hours to 3 days. Two patients in this study developed cardiac arrest. Low HR and hypotension improved in most patients 3 to 5 days after decompression.

The FVC on admission and at discharge was available for 62 (63.9%) and 75 patients, respectively. The mean FVC on admission and at discharge was 1.34 ± 0.46 L and 2.21 ± 0.41 L (*P* < .0001), respectively. Six patients underwent tracheostomy within 3 days postoperatively, including one patient in whom brain death occurred. Eleven patients underwent emergent intubation and received ventilator support for 1 to 5 days in the intensive care unit (ICU). The other patients maintained a pulse oxygen saturation (SpO2) above 95% through supplemental oxygen use. Five patients developed pneumonia, as confirmed by lung CT and etiological examination.

## 4 Discussion

This study provides novel information regarding the influence of early extensive posterior decompression on these acute stage complications for patients with severe tcSCI. We found a significant reduction in the rate of hyponatremia, hypotension, and tracheotomy in severe tcSCI patients undergoing extensive posterior decompression within 24 h by promoting recovery of spinal cord function.

Numerous previous clinical studies on tcSCI have suggested that early surgical (<24 h, even <8 h) decompression might promote neurological recovery.^[13–16]^ In this study, we combined early surgical (<24 h) intervention and extensive posterior surgical decompression for severe tcSCI patients and found a significant increase in the rate of AIS one-grade conversion (80.4%) at discharge. This AIS conversion rate was significantly higher than that (59.5%) at 6 months in a study by Jug et al who used early anterior discectomy (ADF) or corpectomy (ACF) and fusion surgery within 0 to 24 h after injury.^[17]^ The results suggest that early extensive posterior decompression significantly promotes neurological recovery.

In addition, we mainly analyzed the influence of early extensive decompression on acute stage complications in patients with severe tcSCI. It is known that hyponatremia and hypotension associated with sympathetic dysfunction are major complications in addition to sensory and motor deficits during the acute stage after tcSCI.^[1,2]^ These have been shown to be associated with poorer outcomes, such as secondary SCI, pulmonary edema and, rarely, cardiac arrest^[5,6,18]^; thus, they must be emphasized in the clinic. Nakao reported that 78 (45.3%) subjects showed hypotension and 86 (50%) showed hyponatremia in a single institution study that 172 cervical SCI patients with 77.3% cases underwent surgery but did not indicate the surgical procedures.^[3]^ In addition, Furlan et al reported that hyponatremia occurred in 85.7% of patients after tcSCI.^[1]^ Popa reported that 68% of motor-complete-cervical SCI developed hypotension.^[19]^ In this study, we found that the incidence of hyponatremia (41.2%) and hypotension (13.4%) in tcSCI patients who underwent early extensive posterior decompression surgery was significantly lower than that in other studies,^[1,3,19]^ and these complications could be corrected early. Our results suggest that early extensive decompression can significantly reduce the incidence of hyponatremia and hypotension, possibly by restoring descending sympathetic circuits as soon as possible.

Furthermore, our results demonstrate that early extensive decompression can significantly reduce the complications of tracheotomy (n = 6; 6.2%) and pulmonary infection (n = 5; 5.2%) and promote early pulmonary function recovery (FVC at discharge: 2.21 ± 0.41 L vs on admission: 1.34 ± 0.46 L). However, in a previous study that did not indicate the surgical procedures, 58 out of 354 patients (16.4%) underwent tracheotomy.^[20]^ In a single-center retrospective study, Aarabi even implemented a planned tracheotomy for all patients with a high cervical injury.^[21]^ In a study of early posterior laminectomy decompression for tSCI (perhaps not extensive decompression), Kreinest reported that pulmonary infection (30.8%) was the most common complication after urinary tract infection (57.9%).^[22]^ In this study, one potential reason for the reduced rate of respiratory complications may be early extensive decompression leading to early respiratory muscle function recovery, simultaneously avoiding tissue edema and expectoration obstruction caused by tracheal traction in surgery with an anterior approach.

### 4.1 Limitations

Our study has some limitations. First, our study lacked a comparison between conservative treatment and surgical treatment by anterior or combined posterior and anterior approaches. Second, we did not eliminate the effects of drinking water, sodium intake and dexamethasone on blood sodium. Finally, we did not analyze the influence of a history of hypertension and heart disease on BP and HR.

## 5 Conclusion

Our retrospective analysis results suggest that early expansive posterior surgery decompression significantly reduces the incidence of hyponatremia, hypotension, and tracheotomy complications by promoting recovery of spinal cord function after severe tcSCI. Larger, prospective controlled studies are needed to validate these findings.

## Acknowledgments

We thank Dr. Yongshu Lan for technical assistance with imaging.

## Author contributions

Dr. QW was responsible for the study design. Dr. CHY was responsible for performing the experiments, analyzing the data, and drafting the manuscript. Dr. CHY, Dr. GJW and Dr. QW were responsible for revising the manuscript and writing the response letter. Dr. GZL and Dr. SX were responsible for collecting the imaging, and Dr. CHY was responsible for conducting the literature review. All authors have read and approved the final manuscript.
